# Inflammation induced PD-L1-specific T cells

**DOI:** 10.15698/cst2019.10.201

**Published:** 2019-09-13

**Authors:** Shamaila Munir, Mia Thorup Lundsager, Mia Aabroe Jørgensen, Morten Hansen, Trine Hilkjær Petersen, Charlotte Menne Bonefeld, Christina Friese, Özcan Met, Per thor Straten, Mads Hald Andersen

**Affiliations:** 1 National Center for Cancer Immune Therapy (CCIT-dk), Copenhagen University Hospital, Herlev, Denmark.; 2 The LEO Foundation Skin Immunology Research Center, Department of Immunology and Microbiology, University of Copenhagen, Copenhagen, Denmark.; 3 IO Biotech ApS, DK-2200 Copenhagen, Denmark.

**Keywords:** PD-L1, T-cell immunity, Inflammation, IFN-γ, anti-Tregs, anti-regulatory T-cells

## Abstract

PD-L1-specific T cells are a natural part of the T-cell repertoire in humans. Hence, we have previously described spontaneous CD8^+^ and CD4^+^ T-cell reactivity against PD-L1 in the peripheral blood of patients with various cancers as well as in healthy donors. It is well described that the expression of the PD-L1 protein is introduced in cells by pro-inflammatory cytokines, e.g. IFN-γ. In the current study, we were able to directly link inflammation with PD-L1-specific T cells by showing that inflammatory mediators such as IFN-γ generate measurable numbers of PD-L1-specific T cells in human PBMCs as well as in *in vivo* models. These PD-L1-specific T cells can vigorously modulate the cell compartments of the local environment. PD-L1-specific T cells may be important for immune homeostasis by sustaining the ongoing inflammatory response by the suppression of regulatory cell function both directly and indirectly.

## INTRODUCTION

The PD-1/PD-L1 control of T-cell immunity has been underlined by the tremendous success of blocking this pathway in cancer [1, 2]. Thus, it is well described that PD-1 expression on tumor-infiltrating T cells is a major inhibitor of the spontaneous anti-tumor immune response in patients with cancer. PD-1 is a regulatory molecule that delivers inhibitory signals to T cells to make them functionally silent against their antigens. PD-1 and its ligand PD-L1 (B7-H1) play central roles in the formation of an immune-inhibitory tumor microenvironment that protects cancer cells from immune cell–mediated death. PD-L1 is expressed by many different cancer cell types [3–11]. PD-L1 is in addition expressed on antigen-presenting cells, placental cells, and non-hematopoietic cells found in an inflammatory microenvironment as expression of PD-L1 is induced in cells by both type I and II interferons (IFNs)[12, 13]. The immune system consists of many types of regulatory cells that control the strength of the immune response. To maintain the immune balance, these regulatory immune cells (e.g. regulatory T cells (Tregs), M2 macrophages, myeloid-derived suppressor cells (MDSCs), and different dendritic cell subtypes) suppress and terminate immune reactions for example by the expression of PD-L1 [13–16].

Opposed to this, we have described self-reactive, pro-inflammatory T cells, defined as anti-Tregs, that specifically target immune-suppressive cells [17] and counteract the range of counter-regulatory feedback signals including PD-L1. We thus described spontaneous T-cell reactivity against PD-L1 in the peripheral blood of patients with various cancers and in healthy donors [18, 19]. PD-L1–specific T cells reacted towards PD-L1– expressing non-malignant cells in a PD-L1–dependent manner. Additionally, we found that PD-L1–specific T cells killed PD-L1–expressing cancer cells [20–22]. Of note, humoral recognition of PD-L1 has been decribed in rheumatic arthritis [23]. In the current study we sat out to analyze the possible link between inflammatory mediators and PD-L1-specific T-cell immunity.

## RESULTS

### Pro-inflammatory cytokines induce expansion of PD-L1-specific T cells *in vitro*

**Figure 1:**
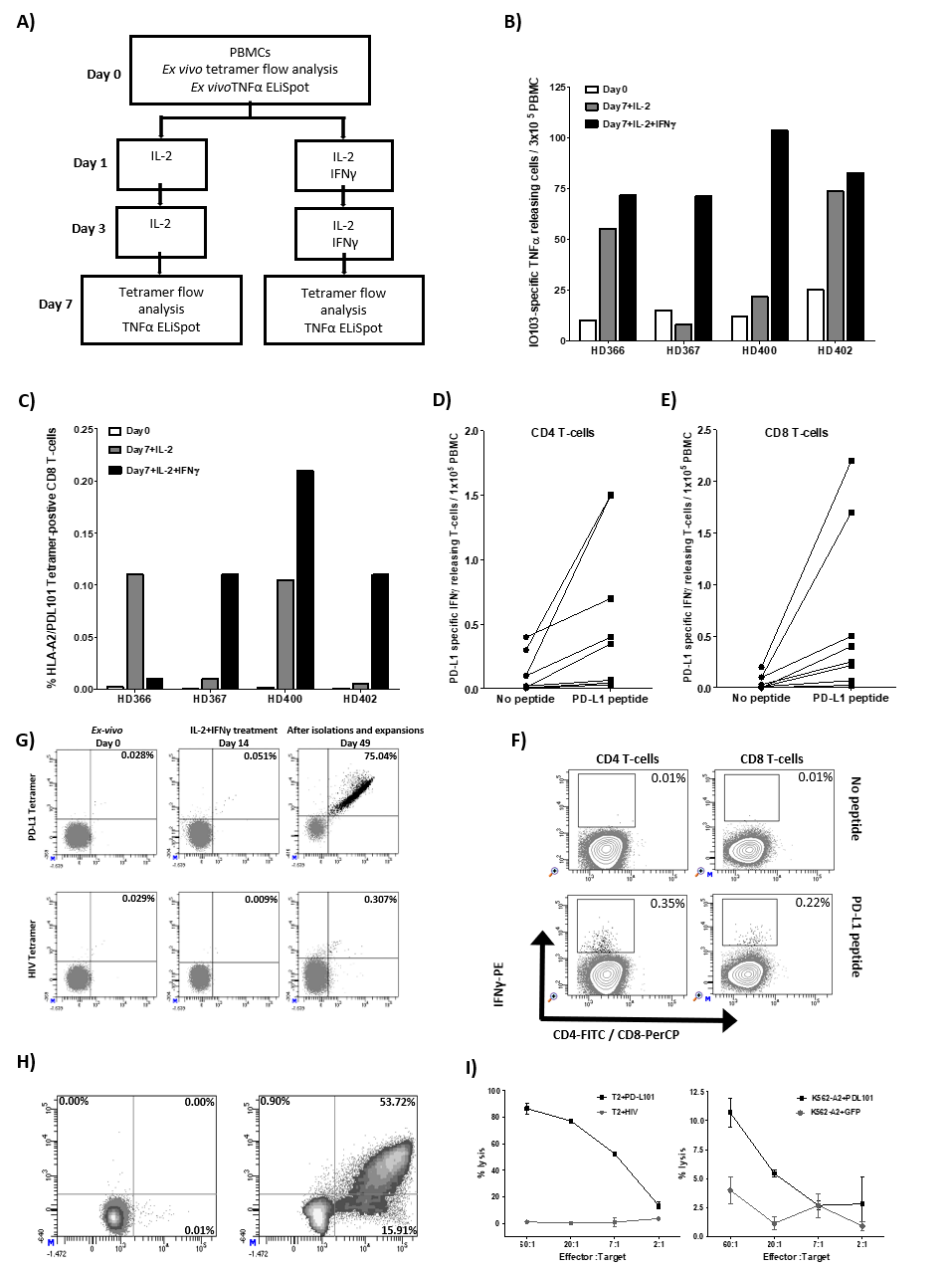
FIGURE 1: Pro-inflammatory cytokines induce expansion of PD-L1-specific T cells *in vitro.* **(A)** Experimental set up: PBMCs from four healthy donors were stimulated twice with IL-2 with or without IFN-γ. On day 7, cultures were examined for T-cell reactivity towards PD-L1 by either tetramer flow analysis or TNF-α-ELISPOT. **(B)** Tetramer analysis of PD-L1-specific CD8 T cells at day 0 (white bars) and at day 7 for cultures stimulated with IL-2 either without IFN-γ (black bars) or with IFN-γ (grey bars). Cells were stained with the tetramers HLA-A2/(PDL1_15-23_; LLNAFTVTV)-PE/APC or HLA-A2/(HIV-1 pol476-484)-PE/APC. **(C)** TNF-α-ELISPOT responses against IO103 (PDL_19-28_; FMTYWHLLNAFTVTVPKDL) peptide at day 0 (white bars) and at day 7 for cultures stimulated with IL-2 either without IFN-γ (black bars) or with IFN-γ (grey bars). All experiments were performed in duplicates and the average number of IO103 induced spots (after subtraction of spots without added peptide) are calculated per 3x10^5^ PBMCs for each donor. **(D, E)** PBMCs from 8 healthy donors were stimulated three times with IL-2 and IFN-γ. On day 7, cells were incubated with and without PD-L1 peptides (PDL1_15-23_ and IO103, PDL_19-28_) for 4 hr. IFN-γ-secreting PD-L1 peptide reactive CD4 and CD8 T-cells were analyzed using IFN-γ secretion assay. **(F)** Example of flow analysis of IFN-γ secreting PD-L1 specific CD4 and CD8 T cells from one healthy donor. **(G)** PBMCs from a healthy donors (HD400) were stimulated three times with IL-2 and IFN-γ. T cells were isolated three times using tetramers HLA-A2/ (PDL1_15-23_; LLNAFTVTV)-PE and anti-PE MACS microbeads. Enriched T cells were expanded using high dose IL-2 (6,000 U/ml). On day 49, enriched T cells were analyzed for PD-L1 specificity by tetramer analysis, intracellular cytokine staining (ICS) and ^51^Cr-release cytotoxicity assay. Tetramer analysis of the resulting T-cell culture at day 0 (*ex vivo*), at day 14 (after three stimulations with IL-2 and IFN-γ), and at day 49 (after isolation and expansion) using the tetramers HLA-A2/(PDL1_15-23_; LLNAFTVTV)-PE/APC, HLA-A2/(HIV-1 pol476-484)-PE. **(H)**On day 49, the resulting T-cell cultures were stimulated for 5 hours either with an irrelevant HIV peptide (HIV-1 pol476-484) or PD-L101 peptide (PDL1_15-23_; LLNAFTVTV) before being analyzed for intracellular IFN-γ/TNF-α staining. **(I)** The resulting T-cell cultures were analyzed on day 49 using ^51^Cr-release cytotoxicity assay. Lysis of TAP-deficient T2 cells (pulsed with PD-L101 (PDL1_15-23_)) or with irrelevant HIV peptide (HIV-1 pol476-484)) (left) or the HLA-A2-transfected leukemic cell line, K562, either transduced with the PD-L1 protein or with GFP (right).

We have previously identified a 19 amino-acid long T-cell epitope, entitled IO103 (PDL1_9-27_; FMTYWHLLNAFTVTVPKDL) that both contains CD4 and CD8 epitopes including the HLA-A2-restricted epitope entitled PDL101 (PDL1_15-23_; LLNAFTVTV). The peptide is part of the signal-peptide sequence of the PD-L1 protein. As it is well described that PD-L1 expression is upregulated in pro-inflammatory environments due to e.g. IFN-γ, we sat out to examine the direct effect hereof on PD-L1-specific T cells. Thus, PBMCs (peripheral blood mononuclear cells) from healthy donors were stimulated twice with IL-2 with and without IFN-γ. On day 7, cultures were examined for reactivity towards IO103 peptide by TNF-α-ELISPOT t as well as HLA-A2/ LLNAFTVTV tetramer by flow analysis (**Figure 1A-C**). In the cultures from all donors we found a detectable number of PD-L1 tetramer-positive CD8 T cells that we stimulated with either IL-2 or with IL-2 and IFN-γ (**Figure 1B**). Similarly, in TNF-α-ELISPOT we were able to detect expansion of IO103-reactive T cells in cultures from all donors stimulated with both IL-2 and IFN-γ (**Figure 1C**). The ELISPOT assays were performed in duplicates and therefore statistical analysis was not performed.

Finally, we examined the presence of PD-L1 specific T cells in PBMC cultures from eight healthy donors that were treated three times with IL-2 and IFN-γ using the IFN-γ-secretion and -detection assay. We describe PD-L1 specific CD4 and CD8 T cells in most of these donors (**Figure 1D-F**).

### PD-L1 specificity and functionally of cytokine-stimulated T cells

Next, we examined if the IFN-γ-induced PD-L1-specific T cells are functional. Thus, we isolated the tetramer positive cells using tetramer MACS beads isolation assay from one of the donors (BC400) and expanded the isolated cells using high dose IL-2 (**Figure 1G**). IFN-γ-induced PD-L1-specific T cells were successfully enriched up to 75.04% (**Figure 1G**). The specificity and functionality of the enriched and expanded cells were subsequently examined. First, we examined the response towards the PDL101 peptide using intracellular staining for IFN-γ and TNF-α. Around 70% of CD8 cells secreted IFN-γ or TNF-α in response to PD-L101 (**Figure 1H**). Next, we examined the cytotoxic capability of the enriched T cells by ^51^Cr release assay. The enriched T cells were able to lyse TAP-deficient T2 cells pulsed with PD-L101 but did not recognize T2 cells pulsed with a negative control peptide from HIV (**Figure 1I**). Furthermore, PD-L1-specific T cells recognized and killed human leukocyte antigen (HLA)-A2-transfected K562 cells, which were transduced with PD-L1 protein but did not recognize PD-L1 negative control HLA-A2-transfected K562 cells (**Figure 1I**). Hence, the IL-2 and IFN-γ cytokine expanded T cells were indeed PD-L1-specific T cells.

### Reduction of Tregs when cultured with IFN-y induced PD-L1 specific T cells

**Figure 2:**
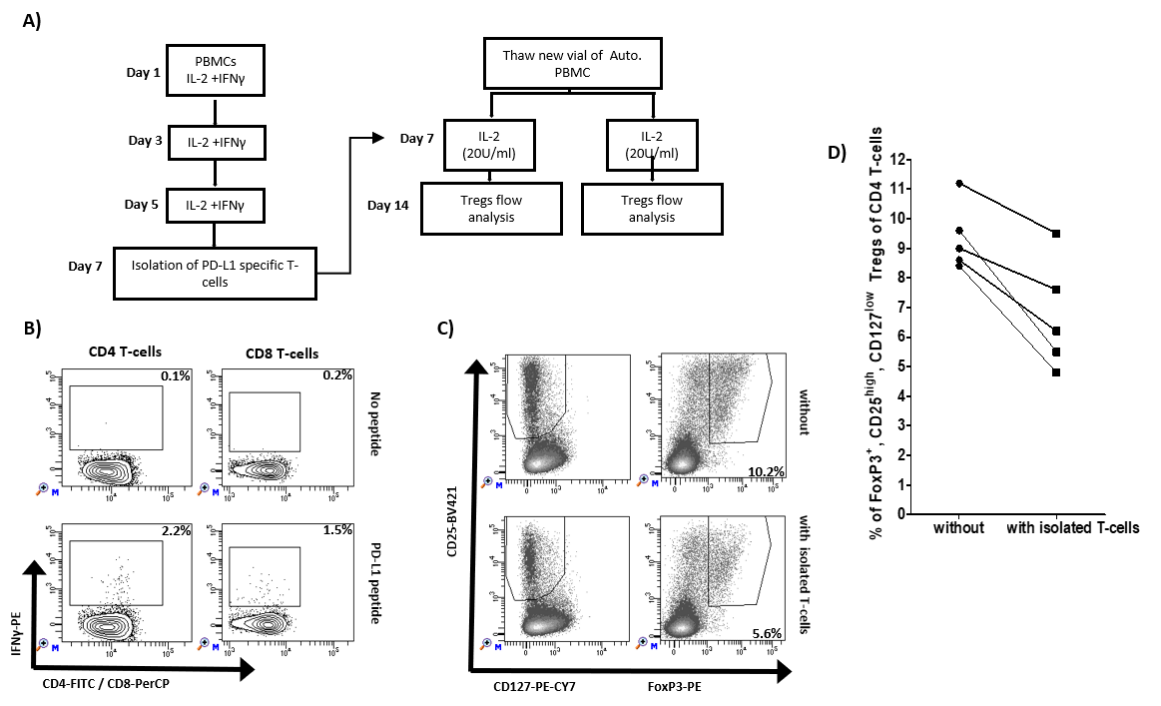
FIGURE 2: Impact of IFN-γ-induced PD-L1-specific T cells on regulatory T cells. **(A)** Experimental set up: PBMCs from five healthy donors were stimulated three times with IL-2 and IFN-γ. On day 7, PD-L1 specific T cells were isolated using IFN-γ secretion and capture assay. The isolated cells were added to freshly thawed autologous PBMCs. On day 14, frequency of Tregs in cultures with or without addition of isolated T cells were analyzed using flow cytometry analysis. **(B)** Example of flow analysis of PD-L1 specific CD4 and CD8 T cells isolation on day 7. **(C)** Example of gating strategy of FoxP3^+^, CD25^high^, CD127^low^ Tregs of CD4 T cells. **(D)** Percentage of FoxP3^+^, CD25^high^, CD127^low^ Tregs of CD4 T-cells from five different healthy donors without and with addition of isolated PD-L1 specific T cells.

Previously, we have described that PD-L1 specific T-cells can augment effector function of T cells. As an example, the addition of the IO103 epitope augments the general T cell response to a dendritic cell–based cancer vaccine [24]. Here, we examined if the inflammation induced PD-L1 specific T cells impact the frequency of Tregs. We isolated IFN-y/IL2-induced PD-L1 specific T cells using IFN-y-secretion and- capture assay and added these to autologus PBMCs from five healthy donors added (**Figure 2A**). After one week, the cultures were examined for the frequency of Tregs. In all five healthy donors, we were able to isolate PD-L1 specific T cells as exemplified in **Figure 2B**. **Figure 2C** shows 5.6% of Foxp3^+^, CD25^high^, CD127^low^ Tregs of CD4 T cells cultured after the addition of isolated PD-L1 specific T cells compared to 10.2% without the addition of isolated T cells. In all five cultures, the percentage of Foxp3^+^ Tregs were reduced after the addition of isolated PD-L1 specific T cells (**Figure 2D**).

### Expansion of PD-L1-specific T cells after IFN-y injections *in vivo*

**Figure 3:**
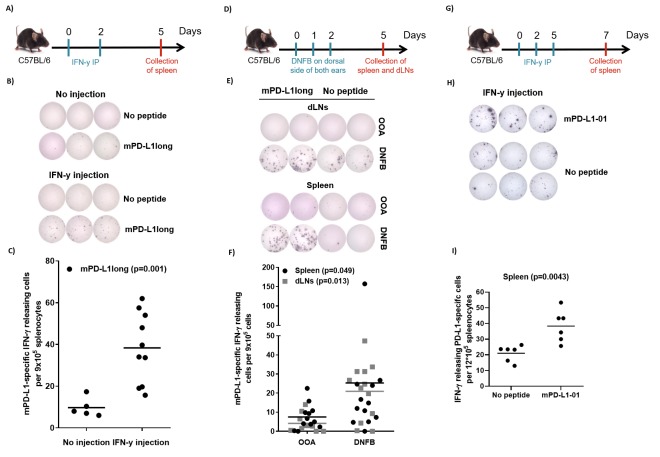
FIGURE 3: Inflammation-induced PD-L1-specific T cells *in vivo*. **(A)** Experimental timeline: C56BL/6 mice were injected twice with 1 µg IFN-y in 200 µl PBS IP (or no injections as a control) on day 0 and day 2. On day 6 the mice were sacrificed, and spleens were analyzed for PD-L1 reactivity. **(B)** Examples of IFN-y-ELISPOT experiments with splenocytes from a control mouse (*top*) or an IFN-y-injected mouse (*bottom*) with or without the mPD-L1long peptide (MRIFAGIIFTACCHLLRA). **(C)** In total, splenocytes from the ten IFN-y-injected mice and the five control mice were examined for reactivity towards the mPD-L1long peptide (MRIFAGIIFTACCHLLRA) by IFN-y- ELISPOT. The average number of mPD-L1long spots (after subtraction of the spots without peptide) was calculated per 9x10^5^ splenocytes for each mouse. Experiments were performed in triplicates. **(D)** Experimental timeline: DNFB or as control OOA was painted on the dorsal side of both ears of C57Bl/6 mice on three consecutive days. On day 5, the mice were sacrificed and spleens and dLNs were removed for further analysis in IFN-y-ELISPOT. **(E)** Examples of IFN-y-ELISPOT experiments with cells from a dLN (*top*) or Spleen (*bottom*) from an OOA-treated control mouse or a DNFB-treated mouse with or without the mPD-L1long peptide (MRIFAGIIFTACCHLLRA). **(F)** In total, cells from dLNs and splenocytes from twelve DNFB-treated mice and twelve OOA-treated control mice were examined for reactivity towards the mPD-L1long peptide (MRIFAGIIFTACCHLLRA) by IFN-y-ELISPOT. The average number of mPD-L1long spots (after subtraction of the spots without peptide) was calculated per 9x10^5^ cells for each mouse. **(G)** Experimental timeline: C56BL/6 mice were injected twice with 1 µg IFN-y in 200 µl PBS IP (or no injections as a control) on day 0,2 and 5. On day 7 the mice were sacrificed, and spleens were analyzed for PD-L1 reactivity. **(H)** Examples of IFN-y-ELISPOT experiments with splenocytes from a control mouse (*top*) or an IFN-y-injected mouse (*bottom*) with or without the short murine peptide, mPD-L1-01 (GIIFTACCHL). **(I)** In total, splenocytes from the six IFN-y-injected mice and the six control mice were examined for reactivity towards the short murine peptide, mPD-L1-01 (GIIFTACCHL) by IFN-y-ELISPOT. The average number of spots with and without mPD-L1-01 (GIIFTACCHL) was counted for each mouse. Experiments were performed in triplicates.

The sequence of human and murine PD-L1 exhibit about 70% similarity. We therefore synthesized a long murine peptide, entitled “mPD-L1long” (PDL11-18, MRIFAGIIFTACCHLLRA), which is located in the same region of the PD-L1 protein as the previously described known human epitope [19]. To examine, if IFN-y may activate PD-L1-specific T cells *in vivo*, we injected ten C56BL/6 mice with 1 µg IFN-y (i.p) twice, two days apart. The mice were sacrificed 3 days after the last injection. Spleens were removed and T-cell specific reactivity against mPD-L1long was analyzed by means of IFN-γ-ELISPOT assay. ELISPOT responses against mPD-L1long were detected in the mice that were injected with IFN-γ (**Figure 3A-C**). **Figure 3B** exemplifies ELISPOT responses against mPD-L1long from two mice with or without IFN-γ injection. The ELISPOT responses against mPD-L1long in the IFN-γ-injected mice were significant compared to the control mice group (P = 0.001; **Figure 3C**).

We next examined if a pro-inflammatory skin response is followed by an increase in naturally occurring PD-L1-specific T-cells. We treated mice with DNFB (2,4-dinitrofluorobenzene), an allergen that is known to induce local inflammation. DNFB was painted on the dorsal side of both ears of 12 C57Bl/6 mice for three consecutive days. On day 5, the mice were sacrificed and spleens and draining lymph nodes (dLNs) were removed and analyzed for PD-L1 reactivity by ELISPOT assay. IFN-γ-ELISPOT responses against mPD-L1long were detected in the group of mice that was treated with DNFB compared to the group of control mice treated with olive oil:acetone mixture (OOA; **Figure 3D-F**). **Figure 3E** exemplifies ELISPOT responses against mPD-L1long using cells from either spleen and dLNs from two mice either treated with OOA or DNFB. The ELISPOT responses against mPD-L1long in the DNFB-treated mice were significant compared to the OOA control mice group both for spleen and dLNs (P = 0.049 and 0.013 respectively; **Figure 3F**).

Due to instabilities of the mPD-L1long peptide during reconstitution, T-cell specific reactivity against a short mPD-L1-01 peptide was also analyzed in splenocytes after three injections with 2 µg IFN-y (intra peritoneal, i.p.) in six male C56BL/6 mice (**Figure 3G**). Significant (Mann-Whitney, P = 0.0043) ELISPOT responses against the short mPDL1 01 peptide were detected compared to the control with no added peptide. (**Figure 3H-I**).

## DISCUSSION

We have previously described circulating, pro-inflammatory PD-L1–specific T cells in healthy donors detectable directly *ex vivo* [18, 19]. Thus, both suppressive as well as effector cells may function mutually as *balance players* for the regulation of the immune system [25]. Immune suppressive counter regulation is well known to be an integrated part of any immune reaction as it controls the strength and magnitude to prevent damage of the host. Counter regulation differs from tolerance in the sense that it is elicited in response to immune activation. PD-L1 plays a central role in the counter regulation of immune responses. It is induced in cells by both type I and II IFNs, which are present at sites of inflammation [26]. Thus, PD-L1 expression is an immune-suppressive feedback signal that is elicited in professional antigen-presenting cells very early in any immune reaction. PD-L1 is therefore highly expressed - even in very potent professional antigen–presenting cells - early during the inflammation process. Although, T cells that are specific to self-antigens require stronger activation signals compared to non–self-specific T cells, they are present at similar frequencies in the blood [27]. The strong activation signal from potent antigen-presenting cells that become PD-L1 positive due to IFN-signaling may be enough to activate PD-L1-specific T cells. Indeed, in the present study we have shown that circulating PD-L1–specific T cells expand as a response to pro-inflammatory mediators. Hence, PD-L1-specific T cells expanded both *in vitro* and *in vivo* as a response to IFN-γ. Likewise, PD-L1-specific T-cell activation could be readily measured in mice that were subjected to DNFB sensitization. DNFB is a well-described contact allergen that induces activation of both CD4^+^ and CD8^+^ allergen-specific effector T cells [28]. Hence, in general PD-L1-specific T cells may function as first responder helper cells at the site of inflammation where they may help responding to infected cells by the release of additional pro-inflammatory cytokines as well as being directly cytolytic towards PD-L1-expressing regulatory cells. This is further substantiated by our previous data showing that the susceptibility of target cells to recognition by PD-L1–specific T cells is increased by pre-incubation with IFN-γ [18]. Additionally, we have previously described that the activation of PD-L1-specific T cells can influence the strength of immune responses by both direct and indirect mechanisms. Hence, we have added PD-L1–specific T cells to cultured PBMCs, one week after stimulating with viral epitopes. The result was an immense increase in the number of virus-specific CD8^+^ T cells *in vitro* [20]. This effect was confirmed in other co-stimulation assays. For example, we observed a significant increase in the numbers of virus-specific T cells in cultures that had been co-stimulated with the PD-L1 peptide epitope, compared to cultures co-stimulated with an irrelevant HIV epitope [29]. These results suggested that PD-L1–specific T cells may support the effector phase of an immune response by removing PD-L1–expressing regulatory immune cells. In the present story, we show that inflammation induced PD-L1 specific T cells indeed influence the number of Tregs when added to unstimulated PBMC cultures. PD-L1-specific T-cells may directly eliminate regulatory immune cells [22] and indirectly augment the effector function of other T cells, i.e. boosting virally or vaccine-triggered immune responses by influencing the immune balance [20, 24, 30].

It is well described that PD-L1 is a key molecule in antagonizing the effects of cancer immunotherapy, moderating the ability to create powerful immune responses against malignant cells. The goal of basically all cancer immunotherapy strategies is to induce immunological activation towards the tumor. Counter-regulatory mechanisms are therefore one of the major complications for the success of cancer immunotherapy. Activation of the already existing PD-L1-specific T-cell response through therapeutic vaccination offers an intriguing way to directly target counter-regulatory pathways in the tumor microenvironment and modulate the local immune suppression without inducing unacceptable toxicity. We have just finalized a phase I, first-in-human PD-L1 based vaccination study (EudraCT no. 2016-000990-19, NCT03042793) including ten patients with the incurable malignancy multiple myeloma. No related adverse reactions above grade II injection-site reactions have occurred and vaccination-induced responses were detected in all patients (Jørgensen *et al.*, in prep.). Vaccination with the PD-L1–derived peptide is additionally being tested in several other ongoing trials, e.g., in combination with an IDO peptide and nivolumab in a phase I/II study in malignant melanoma (NCT03047928) and with an PD-L2 peptide in a phase I study in Follicular Lymphoma (NCT03381768).

The activation of PD-L1-specific T cells through vaccination may suppress immunosuppressive cells and support anti-cancer immune responses like checkpoint inhibitors. A vaccine will, however, not activate all T cells systemically. It will actively recruit T cells to cold tumors and may in addition cause epitope spreading from PD-L1+ tumor cells as PD-L1-specific CD8 T cells directly kill their cognate targets.

## MATERIALS AND METHODS

### Blood Samples

Peripheral blood mononuclear cells (PBMCs) were collected from healthy individuals (average age = 40 years). PBMCs were isolated using Lymphoprep separation, human leukocyte antigen (HLA)-typed, and frozen in fetal calf serum (FCS) with 10% dimethyl sulfoxide (DMSO).

### Mice

C57BL/6 mice were obtained from Taconic M&B (Denmark; IFN-y injection) or Janvier (France; 2,4-dinitrofluorobenzene (DNFB) treatment). All mice were housed in a specific pathogen-free animal facility (Department of Experimental Medicine, Panum Institute, University of Copenhagen, Denmark or National Center for Cancer Immune Therapy, Copenhagen University Hospital at Herlev) and acclimatized for at least one week prior to the initiation of each experiment. Mice entered experiments at ~8–14 weeks of age. The experimental procedures were approved by the national ethics committee on experimental animal welfare and performed according to the Danish guidelines.

### Peptides

A 19 amino acid long polypeptide from the human PD-L1 protein was synthesized (TAG Copenhagen, Copenhagen, Denmark): IO103: PDL1_9–28_, (FMTYWHLLNAFTVTVPKDL). IO103 includes a sequence of 9mer HLA-A2 restricted peptide (entitled “PD-L101”) PDL1_15–23_, (LLNAFTVTV). A long murine PD-L1 peptide, here named mPD-L1long (MRIFAGIIFTACCHLLRA) was synthesized by KJ Ross-Petersen (Denmark; IFN-y injection) or PepScan (Netherlands; DNFB treatment) and a short murine peptide, mPD-L1-01 (GIIFTACCHL) was synthesized by PepScan (Netherlands; IFN-y injection). Human and long murine peptides were dissolved in DMSO in a stock concentration of 10 mM, whereas the short murine peptide was dissolved in acetic acid in a stock concentration of 2 mM.

### IFN-y injection

For screening for PD-L1-specific IFN-y responses towards the mPD-L1long peptide, female mice were injected with 1 µg IFN-y (PeproTech, Sweden) in PBS intra peritoneal (i.p) in a total volume of 200 µl for two times, two days apart and sacrificed 3 days after last injection. Spleens were removed for further analysis by ELISPOT. For screening for PD-L1-specific IFN-y responses towards the mPD-L1-01 peptide, male mice were injected with 2 µg IFN-y (PeproTech, Sweden) in PBS i.p. in a total volume of 200 µl three times, two days apart and sacrificed 3 days after last injection. For the latter two injections, 2400 U of IL-2 (PeproTech) were also injected. Spleens were removed for further analysis by ELISPOT.

### DNFB treatment

Twelve mice were either painted with 25 µl 0.15% DNFB (Sigma, Denmark) in a 1:4 olive oil:acetone (OOA) mixture, or 25 µl OOA as an control, on the dorsal side of both ears for three consecutive days (day 0-2) and sacrificed on day 5. Draining lymph nodes (dLNs) and spleens was removed for further analysis by IFN-γ-ELISPOT.

### Single-cell solution of spleen and draining lymph nodes

Spleen and dLNs from sacrificed mice were removed and smashed through a cell strainer (70 µm) with a syringe plunger and washed with RPMI 1640 with 10% FCS (R10). The red blood cells were removed by red blood cell (RBC) lysis buffer (Qiagen) (1 min) while shaking the tube and then washed another 2 times in R10. The single-cell solution was used for further analysis by IFN-y-ELISPOT

### Induction and enrichment of PD-L1-specific cells

PBMCs from healthy donors were thawed and divided into two cultures. One of the cultures was stimulated with interleukin 2 (IL-2, 120 U/ml, PeproTech) alone and the other with both IL-2 (120 U/ml) and IFN-γ (100 U/ml, PeproTech) on day 1 and day 3. The cultures were analyzed for PD-L1 response using HLA tetramer staining and ELISPOT assay. For the enrichment PD-L1-specific cells, PBMCs from a healthy donor were stimulated with IL-2 (120 U/ml) and IFN-γ (250 U/ml) at day 1, 4 and 7. After two weeks, cells were stained with HLA-A2/PD-L101 tetramer conjugated with PE and subsequently isolated with anti-PE micro beads according to the manufacturing protocol (MACS Miltenyi Biotec). The isolated cells were expanded using REP-media (20 ml of X-VIVO with 5% human serum with 20x10^6^ irradiated feeder cells, 0.6 µg anti-CD3 (eBioscience, clone OKT3) and IL-2 (6,000 U/ml). The enrichment followed by expansion of the PD-L1-specific cells was repeated on day 21 and 35. The enriched PD-L1-specific cells were analyzed for PD-L1 response and functionality using a tetramer-staining assay, intracellular cytokine staining (ICS) and ^51^Cr-release cytotoxicity assay.

### Generation of PD-L1-transduced K562-A2 cells

The cDNA encoding PD-L1 (NM_014143) was synthesized and cloned into a third-generation lentiviral vector pTRP-EGFP (generously provided by Dr. James L. Riley, University of Pennsylvania, Philadelphia, PA) using 5′AvrII/3′SalI restriction sites (GeneArt/ Thermo Fisher Scientific, Regensburg, Germany) generating the lentiviral vector pTRP-EGFP-T2A-PDL1. This vector permits expression of EGFP and PD-L1 from a single RNA transcript. Lentivirus was produced after transfection of 293T human embryonic kidney cells cultured in DMEM (BioWhittaker, Rockville MD, USA), 10% FCS, 100 IU/ml penicillin and 100 µg/ml streptomycin. Cells were seeded at 2.5×10^5^ per well in a 6-well plate 24 h before transfection. For transfection, 1 µg of pTRP-EGFP-T2A-PDL1 and 0.5 µg of packaging and envelope plasmids (pTRP-RSV.Rev, pTRP-GAG-Pol and pTRP-VSVg) was used together with TurboFect Transfection Reagent (Thermo Fisher Scientific). Cells were cultured at humidified atmosphere with 5% CO_2_ for 48 hours before harvesting the viral supernatant. For transduction of K562-A2, cells were incubated with lentivirus supernatant for 72 hours before being sorted using a FACSAria cell sorter (BD Biosciences, San Jose CA, USA). Routine assays for gene expression via flow cytometry and PD-L1-transduced K562-A2 cells were used for experimental analysis as indicated. K562-A2 cells transduced with the lentiviral vector pTRP-EGFP were generated accordingly and used as a control.

### IFN-γ secretion and isolation assay

IFN-γ-secretion assay was used to detect and isolate PD-L1 specific T cells. Briefly, PBMCs from eight different healthy donors were stimulated with both IL-2 (120 U/ml) and IFN-γ (100 U/ml, PeproTech) on day 1, 3 and 5. On day 7, the cultures were examined for reactivity towards IO103: PDL1_9–28_ and PDL101: PDL1_15–23_. According to the protocol provided by MACS Miltenyi Biotec for IFN-γ secretion assay and detection kit, after peptide stimulation the cells were stained with IFN-γ detection antibody for 45 minutes at 37°C, 5% CO_2_ and subsequently stained with IFN-γ capture antibody conjugated with PE and other fluorochrome-conjugated antibodies for surface markers (CD3-BV510, CD8-PerCP, CD4-FITC, all from BD) for 30 minutes at 4°C. Dead cells were stained using LIVE/DEAD® Fixable Near-IR Dead Cell Stain Kit according to manufacturer's instructions. After staining, the PD-L1-specific T cells were analyzed for IFN-γ release using a BD FACSCanto II flow cytometer and BD FACSDiva software.

For the isolation of PD-L1 specific T cells, on day 7, the cells were stained with IFN-γ capture antibody conjugated with PE and subsequently isolated with anti-PE micro beads according to the manufacturing protocol (MACS Miltenyi Biotec). The isolated T cells were either added to freshly thawed autologous PBMCs together with IL-2 (20 U/ml). On day 14, the cultures with and without isolated T cells were analysed for regulatory T cells.

### HLA multimer staining

For multimer/tetramer staining, tetramers coupled with fluorochromes (PE and APC) were prepared using MHC peptide exchange technology. Cells were stained with the HLA tetramer complexes HLA-A2/PD-L101 (PDL1_15–23_; LLNAFTVTV) or HIV-1 (pol_476–484_; ILKEPVHGV) conjugated with APC/PE for 15 minutes at 37 °C. Subsequently, cells were surface-stained with CD3-APC-H7, CD8-PerCP, CD4-FITC and Fixable Viability Stain-510 (all from BD Bioscience) for 30 minutes at 4°C, and analyzed using a BD FACSCanto II flow cytometer and BD FACSDiva software.

### FoxP3+ Treg staining

Five cultures with and without added isolated PD-L1 specific T cells were stained with fluorochrome-conjugated antibodies for surface markers (CD3-FITC, CD4-BV510, CD8-PerCP, CD25-BV421, CD127-PE-Cy7, all from BD) for 30 minutes at 4°C. Dead cells were stained using LIVE/DEAD® Fixable Near-IR Dead Cell Stain Kit according to manufacturer's instructions. After surface staining, cells were washed twice and permeabilized using a standard Fix/Perm kit from eBioscience following the manufacturers' instructions. Following washing with permeabilization buffer, cells were intracellular stained with anti-FoxP3-PE (eBioscience) for 30 minutes at 4°C and and analyzed for FoxP3^+^ Tregs using a BD FACSCanto II flow cytometer and BD FACSDiva software.

### ELISPOT assay

The ELISPOT assay was performed according to the guidelines provided by CIP (http://cimt.eu/cimt/files/dl/cip_guidelines.pdf). Briefly, in murine IFN-y-ELISPOT 96-well MSIPN4W ELISPOT plate (Millipore) were coated with mouse IFN-y-specific capture antibody (AN18; Mabtech) in a concentration of 12 µg/ml in PBS overnight at 4**°**C. Up to 9x10^5^ splenocytes/well were seeded in the plate in two-four replicates. To investigate IFN-y response to various PD-L1 peptides, cells were incubated with different peptides (5 µM) or R10 as control for 18-20 hours at 37**°**C. ELISPOT was developed with mouse IFN-y-specific detection antibody (R4-6A2-biotin; Mabtech) in a concentration of 1 µg/ml in buffer (PBS, 0.5% BSA and NaN_3_) for 2 hours at room temperature, followed by 6 washes in PBS. Next, adding of streptavidin-ALP (1:1000; Mabtech) in buffer for 1 hour at room temperature. Spots were developed by adding substrate solution BCIP/NBT (Mabtech) and stopped by washing in tap water. The spots were counted using the ImmunoSpot Series 2.0 Analyzer (CTL-Immunospot). In human TNF-α- ELISPOT, h-TNF-α-specific capture antibody (TNF3/4, 4 µg/ml, MabTech) and h-TNF-α detection antibody (Biotin TNF-5, 0.25 µg/ml, MabTech) were used.

### Intracellular cytokine staining

For detection of cell subpopulations producing cytokines, PD-L1-specific enriched T cells were stimulated with 5 µg/ml of relevant or irrelevant peptide for 5 hours at 37 °C, 5% CO_2_. Cells were treated with GolgiPlug (BD) at a dilution of 1:200 being added after the first hour of incubation. After 4 additional hours, cells were washed twice with PBS, stained with fluorochrome-conjugated antibodies for surface markers (CD3-APC-H7, CD8-PerCP, CD4-FITC and Fixable Viability Stain-510, all from BD). Cells were washed one additional time and thereafter fixed and permeabilized with Fixation/Permeabilization and Permeabilization Buffer (eBioscience), according to manufacturer's instructions. Cells were subsequently stained with fluorochrome-conjugated antibodies for intracellular cytokines. The following combinations were used: IFN-γ-APC (eBioscience), TNF-α- BV421 (eBioscience) and the stained cells were analyzed using a BD FACSCanto II flow cytometer and BD FACSDiva software.

### Cytotoxicity assay

CTL-mediated cytotoxicity of the PD-L1-enriched cells was measured by ^51^Chromium-release assay as previously described [25]. T2 cells pulsed HIV-1 pol_476-484_ (ILKEPVHGV) or PD-L101 (PDL1_15–23_) or the natural killer target cell line K562 expressing A2 transduced with either PD-L1 protein or GFP were used as target cells. The cell lines included in the study were tested and authenticated by HLA genotyping. The cell lines were routinely examined for their HLA typing and antigen expression by flow cytometry and coculture assays, respectively.

## Abbreviations:

DMSO: – dimethylsulfoxide,
DNFB: – 2,4-dinitrofluorobenzen,
dNL: – draining lymph node,
HLA: – human leukocyte antigen,
IFN: – interferon,
i.p.: – intra peritoneal,
OAA: – olive oil:acetone mixture,
PBMC: – peripheral blood mononuclear cell,
Treg: – regulatory T cell.
